# Bioremediation Potential of a Non-Axenic Cyanobacterium *Synechococcus* sp. for Municipal Wastewater Treatment in the Peruvian Amazon: Growth Kinetics, Ammonium Removal, and Biochemical Characterization Within a Circular Bioeconomy Framework

**DOI:** 10.3390/biotech14020036

**Published:** 2025-05-13

**Authors:** Remy G. Cabezudo, Juan C. Castro, Carlos G. Castro, Hicler N. Rodriguez, Gabriela L. García, Paul M. Vizcarra, Carmen Ruiz-Huamán, Marianela Cobos

**Affiliations:** 1 Environmental Engineering Career, Engineering Faculty, Universidad Peruana de Ciencias Aplicadas (UPC), Lima 15023, Peru; pcigcrui@upc.edu.pe; 2 Specialized Unit of Biotechnology Research Laboratory (UELIB), Natural Resources Research Center of UNAP (CIRNA), Universidad Nacional de la Amazonía Peruana (UNAP), Iquitos 16001, Peru; castrocoboscarlosgilbertox13@gmail.com (C.G.C.); hicler.rodriguez@unapiquitos.edu.pe (H.N.R.); 2160831@unapiquitos.edu.pe (G.L.G.); 3 Academic Department of Biomedical Sciences and Biotechnology, Faculty of Biological Sciences, Universidad Nacional de la Amazonía Peruana (UNAP), Iquitos 16001, Peru; 4 Specialized Unit of Soils Research Laboratory (UELIS), Natural Resources Research Center of UNAP (CIRNA), Universidad Nacional de la Amazonía Peruana (UNAP), Iquitos 16001, Peru; paul.vizcarra@unapiquitos.edu.pe

**Keywords:** biodegradation, circular bioeconomy, environmental remediation, phycocyanin, wastewater

## Abstract

Effective wastewater management is critical for mitigating environmental and health impacts in ecologically sensitive regions like the Peruvian Amazon, where rapid urbanization has led to increased discharge of nutrient-rich effluents into freshwater systems. Conventional treatment methods often fail to address nutrient imbalances while generating secondary pollutants. This study aims to evaluate the bioremediation potential of a non-axenic cyanobacterium, *Synechococcus* sp., isolated from the Amazon Basin, for municipal wastewater treatment within a circular bioeconomy framework. The strain was cultivated in different concentrations of municipal wastewater (25%, 50%, 75%, 100%) from Moronacocha Lake in the Peruvian Amazon to assess growth kinetics, ammonium removal efficiency, and biochemical composition. The cyanobacterium exhibited optimal performance in 25% wastewater, achieving the highest specific growth rate (22.8 × 10^−2^ μ·day^−1^) and biomass increase (393.2%), exceeding even the standard BG-11 medium. This treatment also demonstrated exceptional ammonium removal efficiency (95.4%) and enhanced phycocyanin production (33.6 μg/mg, 56% higher than the control). As wastewater concentration increased, both growth parameters and removal efficiency progressively declined. Biochemical analysis revealed that higher wastewater concentrations resulted in decreased protein content and increased lipid accumulation in the biomass. These findings demonstrate the dual potential of *Synechococcus* sp. for effective wastewater remediation and production of valuable biomass with modifiable biochemical characteristics, offering a sustainable approach for wastewater management in the Peruvian Amazon region.

## 1. Introduction

Effective wastewater management is critical for mitigating the environmental and health impacts of urbanization, particularly in ecologically sensitive regions, such as the Peruvian Amazon. Rapid population growth and inadequate infrastructure have exacerbated the discharge of nutrient-rich effluents into freshwater systems, contributing to eutrophication and loss of biodiversity [1,2]. Conventional wastewater treatments, such as anaerobic oxidation and chemical precipitation [3,4], often fail to address nitrogen and phosphorus imbalances while generating secondary pollutants [3,5,6]. This scenario has spurred interest in biotechnological approaches that align with circular bioeconomy principles by simultaneously addressing pollution control and resource recovery [7].

Phototrophic microorganisms, particularly cyanobacteria, have emerged as promising candidates for wastewater bioremediation due to their ability to assimilate nutrients while producing valuable biomass under relatively low-cost conditions [8,9,10]. Among cyanobacteria, members of the genus *Synechococcus* are particularly noteworthy for their metabolic versatility, environmental tolerance, and potential for industrial applications [11,12]. These unicellular organisms can effectively remove nitrogen and phosphorus from wastewater through direct uptake mechanisms, while simultaneously harvesting light energy to generate biomass rich in high-value compounds, including proteins, lipids, carbohydrates, and bioactive pigments [13,14].

The application of cyanobacteria for wastewater treatment represents a sustainable approach that transforms the conventional linear model of resource consumption into a circular system, where waste streams become valuable inputs for biomass production [15]. This paradigm shift aligns with the principles of a circular bioeconomy [7] and is particularly relevant for developing regions like the Peruvian Amazon. Here, the scarcity of conventional infrastructure is counterbalanced by the abundant sunlight and warm temperatures in the region, which create favorable conditions for phototrophic bioremediation systems.

Despite the growing body of research in cyanobacterial bioremediation, much of the existing literature has predominantly focused on axenic strains cultivated under controlled laboratory conditions. This limitation presents a significant gap in our understanding of the behavior and advantages of non-axenic cultures that are adapted to local environmental conditions, which may be more representative of practical applications in natural systems. Furthermore, the characterization of biomass composition in relation to wastewater cultivation is another area that remains underexplored. Comprehensive analyses are crucial, as the nutrient profiles of wastewater can significantly influence the metabolic pathways of cyanobacteria. Understanding these dynamics is essential for tailoring cyanobacterial biomass for downstream applications, including biofuel production, biofertilizers, and other bioproducts in a circular bioeconomy context [15,16].

The Peruvian Amazon region presents unique challenges for wastewater management due to its distinctive climatic conditions, remoteness, and sensitive ecosystems. Conventional treatment technologies often prove economically unfeasible or environmentally unsuitable for this region, creating an urgent need for alternative approaches that are both effective and contextually appropriate. The integration of cyanobacterial bioremediation systems could potentially address these challenges while supporting local economic development through the production of valuable biomass.

This study aims to evaluate the bioremediation potential of a non-axenic *Synechococcus* sp. strain isolated from the Peruvian Amazon for treating municipal wastewater at varying concentrations. Specifically, we investigate the growth profile, ammonium removal efficiency, biochemical composition, and pigment production of the cyanobacterium under different wastewater treatments. The findings are discussed within a circular bioeconomy framework that considers both environmental remediation and resource recovery, with particular attention to the contextual realities of the Peruvian Amazon region.

## 2. Materials and Methods

### 2.1. Study Area and Sample Collection

Municipal wastewater samples were collected from the primary effluent discharge point of Moronacocha Lake (Iquitos, Loreto Region, northeastern Peru; 3°44′41.89″ S, 73°15′56.65″ W), a tropical freshwater system that receives untreated domestic waste from an urban population of 479,866 (Figure S1). The sampling site was selected due to its role as a critical point of entry for municipal wastewater into the lake, with an estimated maximum daily discharge of 1000 m^3^. Notably, approximately 40% of households in the adjacent urban areas lack formal wastewater infrastructure, contributing to the direct effluent released into the lake.

For the experimental assay, 10 L of a complex wastewater sample was collected from the effluent in pre-cleaned, acid-washed, high-density polyethylene containers at intervals of four hours (0, 4, 8, 12, 16, 20, 24, 28, 32, and 36 h) to ensure representative sampling. The samples were immediately placed on ice in the dark and transported to the laboratory within 30 min to minimize biological activity and photodegradation [17].

### 2.2. Sample Processing 

Upon arrival, the wastewater samples were filtered through Whatman filter paper (grade 1) to remove the suspended solids. This step ensured particle-free effluent for downstream cyanobacterial inoculation, while preserving the dissolved nutrients critical for growth analysis.

### 2.3. Cyanobacterial Strain and Cultivation

The non-axenic cyanobacterium *Synechococcus* sp., sourced from the Peruvian Amazon Native Microalgae Culture Collection (maintained by UELIB-CIRNA, Iquitos, Peru), was selected for its documented adaptability to tropical freshwater ecosystems [18]. Stock cultures were maintained in sterile BG-11 medium (pH 7.4 ± 0.2) under controlled laboratory conditions for 4 weeks before experimentation.

### 2.4. Growth Conditions and Scaling

Cultures were incubated in a climate-controlled growth chamber (25.27 ± 0.06 °C) under a 12:12 h light–dark photoperiod. Illumination was provided by 50-W LED panels (Wellmax^®^, Shanghai, China) with Samsung LED chips, calibrated to deliver a photosynthetic photon flux density of 265 ± 10 μmol photons m^−2^ s^−1^ at the culture surface. To ensure homogeneous growth and gas exchange, the cultures were continuously aerated and agitated at 150 rpm using an orbital shaker (Thermo Scientific MaxQ 6000, Waltham, MA, USA).

A semi-continuous cultivation strategy was employed to scale biomass production: the initial 100 mL cultures were aseptically transferred to 500 mL and subsequently to 1 L vessels in a stepwise manner, with transfers timed to coincide with late-exponential growth phases (optical density at 730 nm [OD_730_] ≈ 0.8–1.2). This scaling protocol minimized physiological stress while ensuring a sufficient cyanobacterial inoculum for subsequent bioremediation experiments.

### 2.5. Experimental Treatments

All bioremediation assays were conducted under the standardized culture conditions described previously (25.27 ± 0.06 °C, 12:12 h light–dark cycle, 265 ± 10 μmol photons m^−2^ s^−1^ continuous aeration, and agitation at 150 rpm) [6,19]. Four experimental groups (wastewater at 25, 50, 75, and 100%) and their respective control groups (without the addition of the cyanobacterial inoculum) were established in triplicate. Ultrapure water was used to dilute the wastewater to 25, 50, and 75%. A BG-11 medium control was also included, which provided a baseline for growth under optimized synthetic conditions.

Erlenmeyer flasks containing 200 mL of BG-11 medium or wastewater at four concentrations were inoculated with 2 mL of mid-exponential growth phase *Synechococcus* sp. culture (equivalent to 6.7 mg of dry cyanobacterial biomass). Daily monitoring over the 7-day experimental period tracked dry cyanobacterial biomass accumulation (μg/mL of culture) and ammonium concentration in the cultures (except in the culture with BG-11 medium)

### 2.6. Growth Kinetics

Cyanobacterial growth was monitored at 24 h intervals over the experimental period. One-milliliter culture aliquots were sampled and centrifuged (10,000× *g* for 5 min at 10 °C). The supernatants were transferred to 1.5 mL microtubes and used to determine the concentration of ammonium. Cyanobacterial biomass obtained after centrifugation was dried at 60 °C for 24 h, weighed, and recorded. Measurements were performed in triplicate to ensure reproducibility and expressed as micrograms of cyanobacterial dried biomass by milliliter of culture (μg of cdb/mL). Specific growth rates and percentage of cdb increases were estimated according to [20] using Equations (1) and (2), where *X*_0_ and *X*_7_ represent the cdb/mL at days 0 and 7, respectively:

(1) $$\mathrm{Specific}\;\mathrm{growth}\;\mathrm{rate}\;({\mu \cdot day}^{-1})=\frac{l_{n}(\frac{X_{7}}{X_{0}})}{7}$$

(2) $$\mathrm{Percentage}\;\mathrm{of}\;\mathrm{cdb}\;\mathrm{increase}\;(\%)=\frac{X_{7}-X_{0}}{7}\times 100$$

### 2.7. Ammonium Removal Efficiency

Ammonium concentration was evaluated in each experimental group and their respective control groups at the start (day 0) and every 24 h for seven days. Ammonium concentration was determined via the indophenol blue direct method [21,22], where ammonium ions react with hypochlorite and phenol to form a blue-green chromophore detectable at 640 nm. A standard calibration curve was constructed using analytical grade (>99.9% purity) ammonium chloride (NH_4_Cl). The calibration curve was prepared using at least six concentration points, spanning the expected range of sample concentrations. The linearity of the calibration curve was validated with correlation coefficient (R^2^) exceeding 0.995, ensuring reliable quantification (Figure S2). Quality control standards were analyzed every 10 samples to verify analytical accuracy throughout the analysis period.

The ammonium removal efficiency (RE) was determined using Equation (3), where $S_{0}$ and $S_{7}$ represent the ammonium concentrations in μg·mL^−1^ on days 0 and 7, respectively:

(3) $$\mathrm{RE}\;(\%)=\frac{S_{0}-S_{7}}{S_{0}}\times 100$$

### 2.8. Biochemical Characterization

At the end of the bioremediation experiments (day 7), the total cyanobacterial biomass was harvested by centrifugation and dried, as previously described. From five to 10 mg of cyanobacterial dried biomass (cdb) were used for biochemical analysis.

Ash, lipids, carbohydrates, and proteins were quantified according to the methods described by Cobos et al. [23]. Standard calibration curves for carbohydrate and protein quantification were constructed using analytical-grade (>99.9% purity) glucose and bovine serum albumin (Figure S2).

The photosynthetic pigment content (chlorophyll-a, carotenoids, and phycobiliproteins) was quantified spectrophotometrically using a modified protocol adapted from Cobos et al. [18]. Absorption spectra were recorded using a NanoDrop™ 2000 spectrophotometer (Thermo Fisher Scientific, Waltham, MA, USA) at wavelengths corresponding to pigment-specific absorption maxima: 440 nm and 680 nm for chlorophyll-a, 490 nm for carotenoids (combined β-carotene and xanthophyll absorption), 620 nm (c-phycocyanin, CPC), and 652 nm (allophycocyanin, APC) for phycobiliproteins. Phycocyanin concentrations were calculated using empirically derived Equations (4) and (5) to correct for the spectral overlap between CPC and APC, as defined by Bennett and Bogorad [24]:

(4) $$\mathrm{CPC}\;({mg\;mL}^{-1})=\frac{A_{620}-0.474\;\times \;A_{652}}{5.34}$$

(5) $$\mathrm{APC}\;({mg\;mL}^{-1})=\frac{A_{652}-0.208\;\times \;A_{620}}{5.09}$$

To verify and quantify carotenoids in the cyanobacterial biomass, we conducted spectrophotometric analysis using a chloroform solution of analytical-grade β-carotene (C4582, Sigma-Aldrich (St. Louis, MO, USA), >95% purity) at a concentration of 50 mg/mL as a reference standard (Figure S3). The absorption spectra of the extracted carotenoids from experimental samples were compared against this standard to confirm their presence and relative abundance.

For phycocyanin characterization, we performed partial purification of these pigment-proteins from 10 g of fresh cyanobacterial biomass harvested during mid-exponential growth phase. The purification process followed the protocol described by Patel et al. [25], which involved ammonium sulfate precipitation followed by dialysis (Figure S4). This procedure allowed us to isolate phycocyanins from the cellular matrix while maintaining their structural integrity for subsequent spectral analysis. The partially purified phycocyanin exhibited the characteristic absorption peaks, confirming the presence of these valuable pigment-proteins in the *Synechococcus* sp. culture.

### 2.9. Statistical Analysis

Statistical analyses were conducted to evaluate the differences in growth kinetics, ammonium removal efficiency, and biochemical composition across the experimental groups. Normality and homogeneity of variances were first assessed using the Anderson-Darling test (α = 0.05) for all variables.

A two-way ANOVA (factors: treatment × time) with Tukey’s post hoc test was applied to analyze differences in *Synechococcus* sp. growth profile over the 7-day experimental period. This model assesses both temporal dynamics and treatment-specific effects.

For endpoint measurements (day 7), parametric data meeting normality assumptions were analyzed using one-way analysis of variance (ANOVA) followed by Tukey’s multi-comparison test. Non-parametric datasets were evaluated using the Kruskal–Wallis test with Dunn’s post hoc correction. Pairwise comparisons between specific treatments were conducted using the Mann–Whitney U test with Bonferroni adjustment for family wise error rate control.

All analyses were performed using GraphPad Prism 10.0 (GraphPad Software, San Diego, CA, USA), with statistical significance defined at α = 0.05. Results are reported as the average ± SD unless otherwise noted.

## 3. Results and Discussion

### 3.1. Growth Kinetics

**Figure 1:**
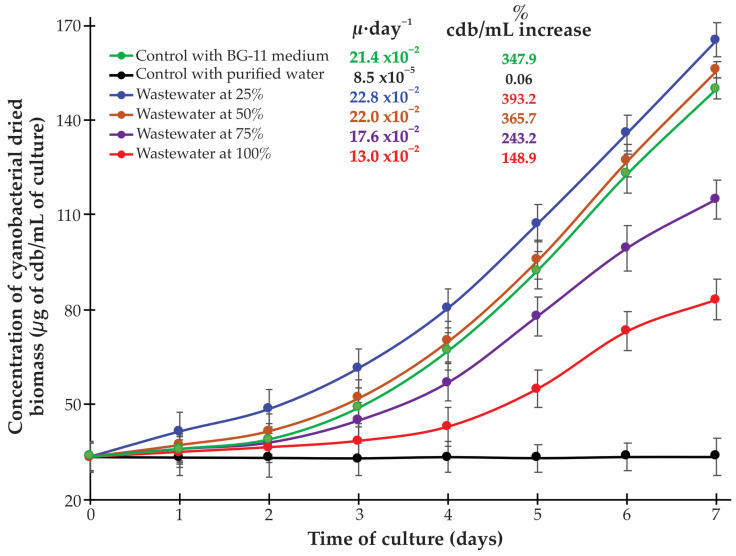
Growth kinetics of *Synechococcus* sp. cultivated in different concentrations of municipal wastewater over a 7-day period. The graph displays the concentration of dry cyanobacterial biomass (μg/mL of culture) for each treatment condition. Specific growth rates (μ⋅day^−1^) and percentage of cyanobacterial dried biomass increase are indicated in the legend for each treatment. The highest growth performance was observed in 25% wastewater (22.8 × 10^−2^ μ⋅day^−1^, 393.2% increase), exceeding even the standard BG-11 medium (21.4 × 10^−2^ μ⋅day^−1^, 347.9% increase). Growth parameters progressively decreased with increasing wastewater concentration, while the purified water control showed minimal growth (8.5 × 10^−5^ μ⋅day^−1^, 0.06% increase). Error bars represent standard deviation (n = 3).

The growth in *Synechococcus* sp. was successfully established across all tested wastewater concentrations, with quantifiable differences in growth kinetics and biomass accumulation between treatments (Figure 1). The specific growth rate (μ⋅day^−1^) and percentage increase in biomass concentration revealed notable patterns that provide insight into the optimal conditions for cyanobacterial cultivation in municipal wastewater from the Peruvian Amazon region. *Synechococcus* sp. exhibited its highest specific growth rate (22.8 × 10^−2^ μ⋅day^−1^) and greatest biomass increase (393.2%) in 25% wastewater, surpassing even the performance in the standard BG-11 medium (21.4 × 10^−2^ μ⋅day^−1^, 347.9% increase).

A clear inverse relationship was observed between wastewater concentration and growth parameters. As wastewater concentration increased from 25% to 100%, both specific growth rate and percent biomass increase progressively declined. The 50% wastewater treatment maintained a growth rate (22.0 × 10^−2^ μ⋅day^−1^) and biomass increase (365.7%) comparable to the BG-11 control, while the 75% and 100% treatments showed substantially reduced performance (17.6 × 10^−2^ μ⋅day^−1^ with 243.2% increase, and 13.0 × 10^−2^ μ⋅day^−1^ with 148.9% increase, respectively). This pattern suggests that while wastewater from Moronacoha Lake provides essential nutrients for cyanobacterial growth, certain components may exert concentration-dependent inhibitory effects, as similarly observed by several investigations [9,19,26,27]. However, these findings have important implications for practical applications, as they suggest that a fed-batch or semi-continuous cultivation system with appropriate dilution could optimize biomass productivity while simultaneously treating wastewater, as was demonstrated in recent publications [28,29,30,31,32].

Another potential strategy for practical implementation could involve a multi-stage cultivation approach as suggested by Sutherland et al. [33], where initial treatment uses diluted wastewater to maximize biomass productivity, followed by subsequent stages with progressively higher wastewater concentrations to achieve comprehensive nutrient removal while maintaining reasonable growth rates.

But it is important to take into account that the relationship between nutrient stoichiometry and biomass productivity has significant implications for large-scale applications. Anaerobically digested wastewater, characterized by high ammonium and orthophosphate content, could also serve as a cost-effective growth medium for *Synechococcus* cultivation when maintained below inhibitory thresholds [34]. Furthermore, wastewater nutrient ratios influence not only growth rates but also cellular stoichiometry, which directly impacts the nutrient cycling capacity of the cyanobacteria and bioremediation efficiency [35].

### 3.2. Ammonium Removal Efficiency

**Figure 2:**
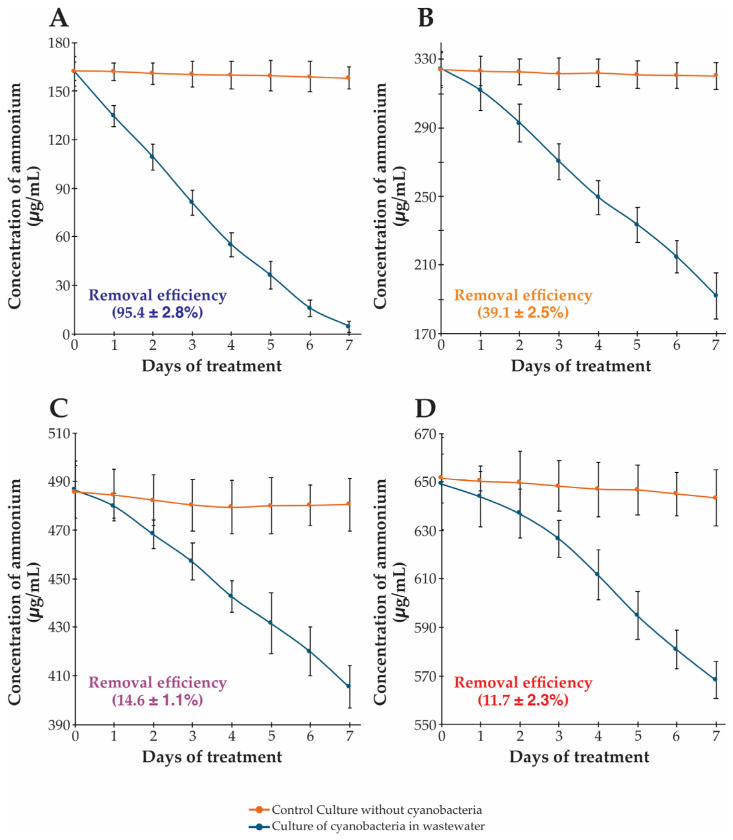
Ammonium removal by *Synechococcus* sp. in different wastewater concentrations over a 7-day treatment period. (**A**) 25% wastewater with initial ammonium concentration ~160 μg/mL, resulting in 95.4 ± 2.8% removal efficiency; (**B**) 50% wastewater with initial concentration ~330 μg/mL, resulting in 39.1 ± 2.5% removal efficiency; (**C**) 75% wastewater with initial concentration ~490 μg/mL, resulting in 14.6 ± 1.1% removal efficiency; (**D**) 100% wastewater with initial concentration ~650 μg/mL, resulting in 11.7 ± 2.3% removal efficiency. Red lines represent control cultures without cyanobacteria; blue lines represent cultures with cyanobacteria. Error bars represent standard deviation (n = 3). Note the inverse relationship between wastewater concentration and removal efficiency, with optimal performance at the 25% wastewater dilution.

The bioremediation capacity of *Synechococcus* sp. was evaluated by quantifying its ammonium removal efficiency in municipal wastewater across different dilution levels and corresponding initial ammonium concentrations (Figure 2). The cyanobacterial cultures exhibited consistent ammonium reduction throughout the 7-day experimental period, while control cultures maintained stable levels, confirming that the observed removal was biologically mediated.

In 25% wastewater treatment (initial ammonium = 162.3 ± 9.3 μg/mL), *Synechococcus* sp. demonstrated exceptional performance with a removal efficiency of 95.4 ± 2.8%, achieving near-complete ammonium removal (Figure 2A). This high efficiency coincided with optimal growth rates and biomass production (Figure 1), suggesting favorable conditions for nutrient assimilation and metabolic activity. At higher wastewater concentrations, removal efficiency declined proportionally. In 50% wastewater (324.3 ± 9.7 μg/mL), efficiency decreased to 39.1 ± 2.5% (Figure 2B). Further reductions occurred in 75% (14.6 ± 1.1%) and 100% of wastewater (11.7 ± 2.3%; Figure 2C,D), relating to growth inhibition patterns (Figure 1). This inverse relationship between wastewater concentration and, therefore, ammonium concentration and removal efficiency parallels the growth inhibition pattern observed in Figure 1, providing strong evidence that optimal bioremediation performance occurs under diluted conditions.

The removal of ammonium in non-axenic cyanobacterial cultures appears to be governed by a complex interplay of direct assimilation and bacterial nitrification processes. Cyanobacteria are known to directly assimilate ammonium into their biomass, a process that has been observed to function in synergy with nitrifying bacteria that oxidize ammonium to nitrite and nitrate [36,37]. It was demonstrated that microbial cultures in municipal wastewater can exhibit high rates of both ammonium assimilation and nitrification, emphasizing the potential role of associated bacteria in these mixed communities [36].

The reduced efficiency of ammonium removal at higher substrate concentrations may be attributed to substrate inhibition and physiological stress imposed by co-occurring wastewater pollutants. Under high ammonium conditions, cyanobacteria often exhibit altered photophysiological behavior to mitigate the risk of photodamage by modulating their light-harvesting capacities and protective pigment composition [38]. When cyanobacteria are supplied with ammonium as the primary nitrogen source, there is a pronounced shift in metabolic pathways geared toward photoprotection, including modifications in pigment synthesis and energy dissipation mechanisms. Such changes can be directly correlated with declines in growth rates, as the reallocative stress responses impede the normal assimilation processes [38].

Additionally, the interplay between ammonium concentration and other wastewater components further complicates the metabolic balance within the culture. It was observed that ammonia-rich environments could trigger significant changes in the nitrogen sensing and assimilation pathways at the proteomic level, affecting both biomass production and nutrient removal efficiency [39]. This aligns with observations where higher loads of ammonium, possibly compounded by other contaminants, result in altered biochemical composition and pigment production, ultimately corroborating the hypothesis of substrate inhibition and stress-induced metabolic adjustments [38,39].

Together, these studies illustrate that while simultaneous direct assimilation and nitrification play crucial roles in ammonium removal within non-axenic cyanobacterial systems, environmental factors and the intricacies of intracellular metabolic regulation under high-ammonium conditions can lead to reduced treatment efficiency. The cumulative evidence emphasizes the need for a balanced nutrient load to maintain optimal metabolic function and highlights the complex dynamics that govern wastewater treatment processes in phototrophic cultures.

### 3.3. Biochemical Characterization

**Figure 3:**
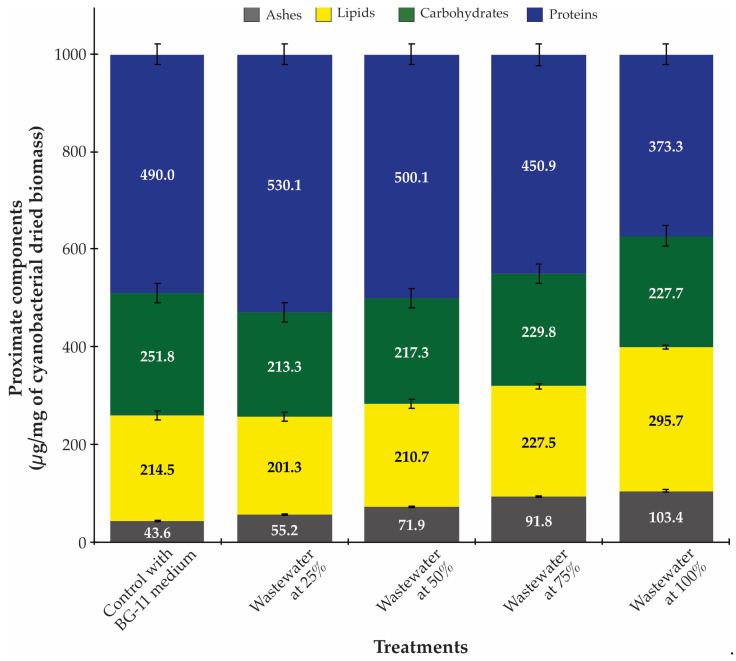
Proximate biochemical composition of *Synechococcus* sp. dried biomass cultivated in different wastewater concentrations. The stacked bars show the content of proteins (blue), carbohydrates (green), lipids (yellow), and ashes (gray) expressed as μg/mg of cyanobacterial dried biomass. Error bars represent standard deviation (n = 3). Note the inverse relationship between protein and lipid content with increasing wastewater concentration, with protein content decreasing and lipid content increasing at higher wastewater concentrations.

The wastewater composition significantly influenced the proximate biochemical profile of the harvested *Synechococcus* sp. biomass (Figure 3). The protein content, which constituted the largest fraction of biomass across all treatments, exhibited a notable decrease with increasing wastewater concentration, from 530.1 μg/mg in 25% wastewater to 373.3 μg/mg in 100% wastewater. This trend may reflect cellular responses to stress conditions at higher wastewater concentrations, as protein synthesis is often downregulated under various environmental stressors [40,41].

In contrast, lipid content displayed a marked increase with increasing wastewater concentration, reaching its maximum of 295.7 μg/mg in 100% wastewater, a 38% increase compared to the BG-11 control (214.5 μg/mg). This enhanced lipid accumulation represents a potentially valuable outcome from a biorefinery perspective, as lipid-rich biomass has applications in biofuel production and high-value nutraceuticals [23,42]. Carbohydrate content showed more complex variation, initially decreasing in 25% wastewater compared to the BG-11 control, but then increasing at higher wastewater concentrations. This non-linear response suggests complex metabolic adaptations to the changing nutrient profiles and potential stressors in the wastewater treatments. The inverse relationship between protein and lipid content aligns with observations by other researchers [2,43,44,45,46,47], who described the plasticity in the metabolism of proteins, lipids, and carbohydrates under nutrient stress and changing environmental conditions in various cyanobacterial and microalgal species.

The ash content observed in the dried biomass of cyanobacteria exhibited a significant increase correlated with wastewater concentration, rising from 43.6 μg/mg in the BG-11 control to 103.4 μg/mg in a 100% wastewater condition. This phenomenon can be attributed to the accumulation of inorganic ions derived from the wastewater matrix. In this context, the ash content in cyanobacteria cultured in different concentrations of wastewater can vary significantly due to the nutrient composition and concentration of the wastewater. Cyanobacteria, being efficient in nutrient uptake, can alter their biochemical composition, including ash content, based on the availability of nutrients and heavy metal content in the wastewater. Studies indicate that the concentration of wastewater influences the growth and biochemical composition of cyanobacteria, which in turn affects the ash content [48,49,50].

**Figure 4:**
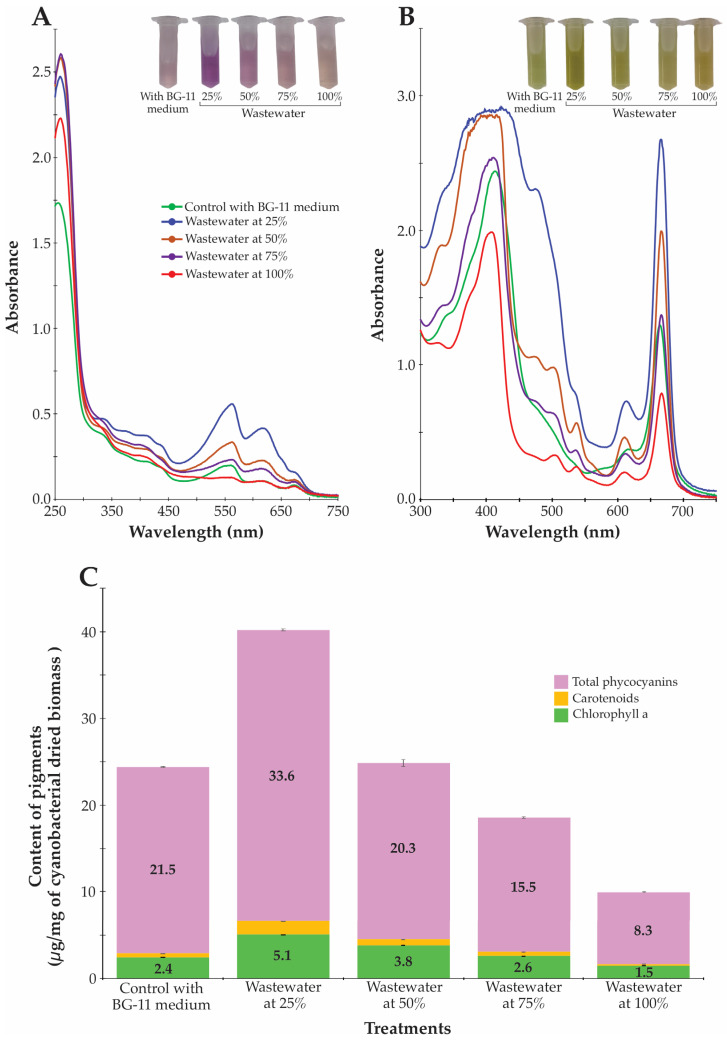
Spectral characteristics and pigment composition of *Synechococcus* sp. cultivated in different wastewater concentrations. (**A**) UV-visible absorption spectra (250–750 nm) with inset showing culture tube coloration across treatments. (**B**) Visible light absorption spectra (300–750 nm) highlighting peaks associated with photosynthetic pigments. (**C**) Content of photosynthetic pigments including total phycocyanins (purple), carotenoids (orange), and chlorophyll a (green) expressed as μg/mg of cyanobacterial dried biomass. Error bars represent standard deviation (n = 3). Note the enhanced phycocyanin production in 25% wastewater treatment compared to other conditions.

The spectrophotometric analysis and quantification of photosynthetic pigments revealed significant adaptations in the *Synechococcus* sp. pigment apparatus in response to wastewater cultivation (Figure 4 and Figure S4). The most striking finding was the enhanced production of phycocyanin, a high-value blue protein pigment with applications in food coloring, cosmetics, and pharmaceuticals [51], in the 25% wastewater treatment, which reached 33.6 μg/mg, representing a 56% increase compared to the BG-11 control (21.5 μg/mg). The absorbance spectrum (Figure 4A and Figure S4) corroborated the phycocyanin quantification results, with the 25% wastewater treatment exhibiting higher absorption peaks in the characteristic phycocyanin regions (550–650 nm). The visual color differences in the microtubes with extracted phycocyanins further confirmed these spectroscopic findings, with more intense blue coloration in the 25% wastewater culture compared to other treatments.

However, all pigments (phycocyanins, carotenoids, and chlorophyll a) showed progressive decreases as wastewater concentration increased beyond 25%, reaching minimum values in the 100% wastewater treatment (Figure 4A–C). Other investigations have also shown that the concentration of phycocyanin and other photosynthetic pigments in cyanobacteria cultures can vary based on the concentration of wastewater used in the culture medium. This variation is influenced by the availability of nutrients, particularly nitrogen and phosphorus, which are abundant in wastewater. For instance, in cultures of *Synechococcus* sp., phycocyanin content was found to vary according to nitrogen levels, while carotenoid content remained stable regardless of wastewater composition [13]. Similarly, the presence of nutrients such as ammonium and phosphate in swine wastewater supports the growth and influences the phycocyanin production in *Thermosynechococcus* sp. CL-1 [52].

The observed pattern indicates that moderate dilution of wastewater (25%) creates optimal conditions for the biosynthesis of pigments in the cyanobacteria *Synechococcus* sp. At this dilution level, the balance of nutrients appears to favor pigment production, enhancing the metabolic pathways associated with pigment biosynthesis. However, as the concentration of wastewater increases beyond this moderate level, the cyanobacteria may experience stress conditions that lead to a redirection of metabolic resources. Elevated concentrations of certain toxic substances or nutrient imbalances can trigger defensive strategies in cyanobacteria, resulting in reduced pigment synthesis. This shift in metabolic focus is well-documented; for instance, Allen and Smith noted that cyanobacteria under nitrogen-stressed conditions may tap into pigment reserves and reutilize nitrogen-rich compounds to sustain growth, thereby compromising pigment production [53].

Additionally, toxic byproducts typically present in higher concentrations of wastewater can inhibit the metabolic pathways involved in pigment biosynthesis. Under high-stress conditions, such as those induced by nutrient limitations or toxic ion exposure, cyanobacteria prioritize growth and survival over the synthesis of secondary metabolites, including pigments [53]. Furthermore, the iron homeostasis and nutrient regulation in cyanobacterial systems are critical, indicating that disturbances in nutrient availability could further detract from optimal pigment production [54]. Thus, while moderate wastewater concentration fosters pigment biosynthesis through nutrient abundance, elevated levels may detrimentally affect cellular processes, illustrating a complex interplay between environmental stressors and metabolic allocation in cyanobacteria.

## 4. Conclusions

This study demonstrates the effective bioremediation potential of a non-axenic *Synechococcus* sp. strain for treating municipal wastewater from the Peruvian Amazon, while simultaneously producing valuable biomass with modifiable biochemical characteristics. The cyanobacterium exhibited robust growth across all wastewater concentrations tested, with performance in diluted wastewater (25%) exceeding even the standard BG-11 medium. This finding is particularly significant as it indicates that municipal wastewater can serve not merely as a substrate for remediation but as an optimized growth medium when appropriately diluted.

The substantial ammonium removal capacity demonstrated across various initial concentrations highlights the potential of this *Synechococcus* strain for addressing one of the primary nutrient concerns in municipal wastewater. Complete removal at lower concentrations and significant reductions at higher concentrations suggest that a multi-stage treatment approach could maximize remediation efficiency while producing biomass with different characteristics at each stage.

The modulation of biochemical composition in response to different wastewater concentrations presents opportunities for tailored biomass production targeted toward specific applications. The enhanced lipid accumulation at higher wastewater concentrations could be particularly valuable for biofuel applications, while the increased phycocyanin production in 25% wastewater opens possibilities for high-value pigment extraction within an integrated biorefinery concept.

The non-axenic nature of the cyanobacterial culture likely contributed to its robust performance across different wastewater conditions, suggesting that engineered consortia may offer advantages over axenic cultures for real-world applications. This approach aligns with emerging understanding that microbial community interactions can enhance resilience and functional performance in bioremediation systems.

Several limitations of this study should be acknowledged. The 7-day experimental period, while sufficient to demonstrate bioremediation potential, may not fully capture long-term performance and stability. Additionally, while ammonium removal was comprehensively evaluated, other important wastewater parameters such as phosphorus, organic carbon, and potential micropollutants warrant further investigation.

Future research should explore continuous or semi-continuous operational modes that better approximate real-world treatment scenarios. The optimization of hydraulic and solid retention times, along with investigations into seasonal variations and scaling considerations, would provide valuable insights for practical implementation. Furthermore, detailed economic and life cycle analyses would help quantify the sustainability benefits of the proposed approach within a circular bioeconomy framework.

In conclusion, this study provides compelling evidence for the potential of *Synechococcus* sp. to serve as a cornerstone organism in developing integrated wastewater treatment systems that simultaneously address environmental challenges while generating valuable bioproducts in the Peruvian Amazon region. The findings contribute to the growing body of knowledge supporting the transition from linear to circular approaches in wastewater management, particularly in regions where conventional infrastructure is limited but biological resources are abundant.

## Data Availability

All relevant data are presented within the manuscript and Supplementary Materials. Additional raw data and detailed experimental protocols are available upon request from the corresponding author.

## Supplementary Materials

The following supporting information can be downloaded at: https://www.mdpi.com/article/10.3390/biotech14020036/s1, Figure S1. Geographic location and aerial views of the municipal wastewater discharge point in Moronacocha Lake, Iquitos, Peru. Top: Location of Iquitos city in northeastern Peru and its position relative to the Amazon River. Middle: Satellite image of the urban area showing the wastewater discharge point (03°44′41.89″ S, 73°15′56.65″ W). Bottom: Detailed aerial view and ground-level photograph of the discharge infrastructure where wastewater samples were collected for this study; Figure S2. Standard calibration curves for analytical measurements used in this study. (A) Ammonium chloride calibration curve (indophenol blue method at 640 nm) showing linear relationship (R^2^ = 0.9995) used for quantifying ammonium in wastewater treatments; (B) Bovine serum albumin calibration curve (R^2^ = 0.9979) used for protein content determination in cyanobacterial biomass; (C) Glucose calibration curve (R^2^ = 0.9978) used for carbohydrate content determination in cyanobacterial biomass. Each calibration curve demonstrates high linearity with correlation coefficients exceeding 0.995, ensuring reliable quantification of respective compounds. Linear regression equations and correlation coefficients (R^2^) are shown for each calibration curve; Figure S3. Absorption spectrum of carotenoids extracted from *Synechococcus* sp. biomass. The spectrum shows a characteristic absorbance pattern of carotenoids with an absorption maximum around 330–340 nm and multiple smaller peaks corresponding to vibrational fine structure. This reference spectrum was used for identification and relative quantification of carotenoid content in cyanobacterial biomass grown under different wastewater treatments; Figure S4. Absorption spectra of phycobiliproteins extracted from *Synechococcus* sp. biomass. (A) Full spectral scan (200–800 nm) of partially purified phycocyanin showing characteristic peaks in the 500–650 nm region; (B) Detailed absorption profile of phycobiliproteins in the visible region (450–700 nm) with characteristic absorption maxima at 565 nm and 620 nm, corresponding to phycoerythrin and phycocyanin, respectively. These spectra were used for pigment identification and quantification in cyanobacterial biomass grown under different wastewater treatments.

## References

[1] UNESCO (2020). Water and Climate Change.

[2] Sisman-Aydin G., Simsek K. (2022). Municipal Wastewater Effects on the Performance of Nutrient Removal, and Lipid, Carbohydrate, and Protein Productivity of Blue-Green Algae *Chroococcus turgidus*. Sustainability.

[3] Verma A., Sharma A., Kumar R., Sharma P. (2023). Nitrate Contamination in Groundwater and Associated Health Risk Assessment for Indo-Gangetic Plain, India. Groundw. Sustain. Dev..

[4] Wang Q., Wang X., Hong Y., Liu X., Zhao G., Zhang H., Zhai Q. (2022). Microalgae Cultivation in Domestic Wastewater for Wastewater Treatment and High Value-Added Production: Species Selection and Comparison. Biochem. Eng. J..

[5] Ye S., Gao L., Zhao J., An M., Wu H., Li M. (2020). Simultaneous Wastewater Treatment and Lipid Production by *Scenedesmus* sp. HXY2. Bioresour. Technol..

[6] Wu K., Atasoy M., Zweers H., Rijnaarts H., Langenhoff A., Fernandes T.V. (2023). Impact of Wastewater Characteristics on the Removal of Organic Micropollutants by *Chlorella sorokiniana*. J. Hazard. Mater..

[7] Haider M.N., Liu C.-G., Tabish T.A., Balakrishnan D., Show P.-L., Qattan S.Y.A., Gull M., Mehmood M.A. (2022). Resource Recovery of the Wastewater-Derived Nutrients into Algal Biomass Followed by Its Cascading Processing to Multiple Products in a Circular Bioeconomy Paradigm. Fermentation.

[8] Aditya L., Mahlia T.M.I., Nguyen L.N., Vu H.P., Nghiem L.D. (2022). Microalgae-Bacteria Consortium for Wastewater Treatment and Biomass Production. Sci. Total Environ..

[9] Janpum C., Pombubpa N., Monshupanee T., Incharoensakdi A., In-Na P. (2022). Advancement on Mixed Microalgal-Bacterial Cultivation Systems for Nitrogen and Phosphorus Recoveries from Wastewater to Promote Sustainable Bioeconomy. J. Biotechnol..

[10] Goyal S., Dhanker R., Hussain T., Ferreira A., Gouveia L., Kumar K., Mohamed H.I. (2023). Modern Advancement in Biotechnological Applications for Wastewater Treatment through Microalgae: A Review. Water. Air Soil Pollut..

[11] Pishbin M., Sarrafzadeh M.-H., Faramarzi M.A. (2021). Nitrate and Phosphate Removal Efficiency of *Synechococcus elongatus* Under Mixotrophic and Heterotrophic Conditions for Wastewater Treatment. Iran. J. Sci. Technol. Trans. Civ. Eng..

[12] De Farias Silva C.E., Bertucco A., Vieira R.C., Abud A.K.D.S., Silva Almeida F.B.P.D. (2022). *Synechococcus* PCC 7002 to Produce a Carbohydrate-Rich Biomass Treating Urban Wastewater. Biofuels.

[13] Bellver M., Ruales E., Díez-Montero R., Escolà Casas M., Matamoros V., Ferrer I. (2025). Natural Pigments and Biogas Recovery from Cyanobacteria Grown in Treated Wastewater. Fate of Organic Microcontaminants. Water Res..

[14] Mathimani T., Alshiekheid M.A., Sabour A., Le T., Xia C. (2024). Appraising the Phycoremediation Potential of Cyanobacterial Strains *Phormidium* and *Oscillatoria* for Nutrient Removal from Textile Wastewater (TWW) and Synchronized Biodiesel Production from TWW-Tolerant Biomass. Environ. Res..

[15] Prabha S., Vijay A.K., Paul R.R., George B. (2022). Cyanobacterial Biorefinery: Towards Economic Feasibility through the Maximum Valorization of Biomass. Sci. Total Environ..

[16] Satya A.D.M., Cheah W.Y., Yazdi S.K., Cheng Y.-S., Khoo K.S., Vo D.-V.N., Bui X.D., Vithanage M., Show P.L. (2023). Progress on Microalgae Cultivation in Wastewater for Bioremediation and Circular Bioeconomy. Environ. Res..

[17] Ummalyma S.B., Singh A. (2022). Biomass Production and Phycoremediation of Microalgae Cultivated in Polluted River Water. Bioresour. Technol..

[18] Cobos M., Condori R.C., Grandez M.A., Estela S.L., Del Aguila M.T., Castro C.G., Rodríguez H.N., Vargas J.A., Tresierra A.B., Barriga L.A. (2022). Genomic Analysis and Biochemical Profiling of an Unaxenic Strain of *Synechococcus* sp. Isolated from the Peruvian Amazon Basin Region. Front. Genet..

[19] Sousa J.F., Amaro H.M., Ribeirinho-Soares S., Esteves A.F., Salgado E.M., Nunes O.C., Pires J.C.M. (2024). Native Microalgae-Bacteria Consortia: A Sustainable Approach for Effective Urban Wastewater Bioremediation and Disinfection. Microorganisms.

[20] Richmond A., Hu Q. (2013). Handbook of Microalgae Culture: Applied Phycology and Biotechnology.

[21] American Public Health Association (2017). Standard Methods for the Examination of Water and Wastewater.

[22] Huizenga J.R., Teelken A.W., Tangerman A., De Jager A.E.J., Gips C.H., Jansen P.L.M. (1998). Determination of Ammonia in Cerebrospinal Fluid Using the Indophenol Direct Method. Mol. Chem. Neuropathol..

[23] Cobos M., Paredes J.D., Maddox J.D., Vargas-Arana G., Flores L., Aguilar C.P., Marapara J.L., Castro J.C. (2017). Isolation and Characterization of Native Microalgae from the Peruvian Amazon with Potential for Biodiesel Production. Energies.

[24] Bennett A., Bogorad L. (1973). Complementary Chromatic Adaptation in a Filamentous Blue-Green Alga. J. Cell Biol..

[25] Patel A., Mishra S., Pawar R., Ghosh P.K. (2005). Purification and Characterization of C-Phycocyanin from Cyanobacterial Species of Marine and Freshwater Habitat. Protein Expr. Purif..

[26] Agawin N.S.R., Duarte C.M., Agustí S. (2000). Response of Mediterranean *Synechococcus* Growth and Loss Rates to Experimental Nutrient Inputs. Mar. Ecol. Prog. Ser..

[27] Korosh T.C., Dutcher A., Pfleger B.F., McMahon K.D. (2018). Inhibition of Cyanobacterial Growth on a Municipal Wastewater Sidestream Is Impacted by Temperature. mSphere.

[28] Solís-Salinas C.E., Patlán-Juárez G., Okoye P.U., Guillén-Garcés A., Sebastian P.J., Arias D.M. (2021). Long-Term Semi-Continuous Production of Carbohydrate-Enriched Microalgae Biomass Cultivated in Low-Loaded Domestic Wastewater. Sci. Total Environ..

[29] Bresaola M.D., Morocho-Jácome A.L., Matsudo M.C., de Carvalho J.C.M. (2019). Semi-Continuous Process as a Promising Technique in *Ankistrodesmus braunii* Cultivation in Photobioreactor. J. Appl. Phycol..

[30] Álvarez X., Otero A. (2020). Nutrient Removal from the Centrate of Anaerobic Digestion of High Ammonium Industrial Wastewater by a Semi-Continuous Culture of *Arthrospira* sp. and *Nostoc* sp. PCC 7413. J. Appl. Phycol..

[31] Markou G. (2015). Fed-Batch Cultivation of *Arthrospira* and *Chlorella* in Ammonia-Rich Wastewater: Optimization of Nutrient Removal and Biomass Production. Bioresour. Technol..

[32] García-Cañedo J.C., Cristiani-Urbina E., Flores-Ortiz C.M., Ponce-Noyola T., Esparza-García F., Cañizares-Villanueva R.O. (2016). Batch and Fed-Batch Culture of *Scenedesmus incrassatulus*: Effect over Biomass, Carotenoid Profile and Concentration, Photosynthetic Efficiency and Non-Photochemical Quenching. Algal Res..

[33] Sutherland D.L., Park J., Heubeck S., Ralph P.J., Craggs R.J. (2020). Size Matters—Microalgae Production and Nutrient Removal in Wastewater Treatment High Rate Algal Ponds of Three Different Sizes. Algal Res..

[34] Dutcher A. (2016). Optimization of Growth Conditions for the Model Bacterium *Synechococcus* sp. PCC 7002 for Chemical Production Using Wastewater-Based Media. Master’s Thesis.

[35] Garcia N.S., Bonachela J.A., Martiny A.C. (2016). Interactions between Growth-Dependent Changes in Cell Size, Nutrient Supply and Cellular Elemental Stoichiometry of Marine *Synechococcus*. ISME J..

[36] Leong W.H., Kiatkittipong K., Kiatkittipong W., Cheng Y.W., Lam M.K., Shamsuddin R., Mohamad M., Lim J.W. (2020). Comparative Performances of Microalgal-Bacterial Co-Cultivation to Bioremediate Synthetic and Municipal Wastewaters Whilst Producing Biodiesel Sustainably. Processes.

[37] Stauch-White K., Srinivasan V.N., Camilla Kuo-Dahab W., Park C., Butler C.S. (2017). The Role of Inorganic Nitrogen in Successful Formation of Granular Biofilms for Wastewater Treatment That Support Cyanobacteria and Bacteria. AMB Express.

[38] Yang X., Dong W., Liu L., Bi Y., Xu W., Wang X. (2023). Uncovering the Differential Growth of *Microcystis aeruginosa* Cultivated under Nitrate and Ammonium from a Photophysiological Perspective. ACS EST Water.

[39] Patel A.K., Huang E.L., Low-Décarie E., Lefsrud M.G. (2015). Comparative Shotgun Proteomic Analysis of Wastewater-Cultured Microalgae: Nitrogen Sensing and Carbon Fixation for Growth and Nutrient Removal in *Chlamydomonas reinhardtii*. J. Proteome Res..

[40] Anand J., Arumugam M. (2015). Enhanced Lipid Accumulation and Biomass Yield of *Scenedesmus quadricauda* Under Nitrogen Starved Condition. Bioresour. Technol..

[41] Breuer G., Lamers P.P., Martens D.E., Draaisma R.B., Wijffels R.H. (2012). The Impact of Nitrogen Starvation on the Dynamics of Triacylglycerol Accumulation in Nine Microalgae Strains. Bioresour. Technol..

[42] Arguelles E.D., Laurena A.C., Monsalud R.G., Martinez-Goss M.R. (2018). Fatty Acid Profile and Fuel-Derived Physico-Chemical Properties of Biodiesel Obtained from an Indigenous Green Microalga, *Desmodesmus* sp. (I-AU1), as Potential Source of Renewable Lipid and High Quality Biodiesel. J. Appl. Phycol..

[43] Rodolfi L., Chini Zittelli G., Bassi N., Padovani G., Biondi N., Bonini G., Tredici M.R. (2009). Microalgae for Oil: Strain Selection, Induction of Lipid Synthesis and Outdoor Mass Cultivation in a Low-Cost Photobioreactor. Biotechnol. Bioeng..

[44] Castielli O., De la Cerda B., Navarro J.A., Hervás M., De la Rosa M.A. (2009). Proteomic Analyses of the Response of Cyanobacteria to Different Stress Conditions. FEBS Lett..

[45] Xiong W., Cano M., Wang B., Douchi D., Yu J. (2017). The Plasticity of Cyanobacterial Carbon Metabolism. Curr. Opin. Chem. Biol..

[46] Yalcin D. (2020). Growth, Lipid Content, and Fatty Acid Profile of Freshwater Cyanobacteria *Dolichospermum affine* (Lemmermann) Wacklin, Hoffmann, & Komárek by Using Modified Nutrient Media. Aquac. Int..

[47] Ciebiada M., Kubiak K., Daroch M. (2020). Modifying the Cyanobacterial Metabolism as a Key to Efficient Biopolymer Production in Photosynthetic Microorganisms. Int. J. Mol. Sci..

[48] Singh D.V., Upadhyay A.K., Singh R., Singh D.P. (2020). Eco-Friendly and Eco Technological Approaches in Treatment of Wastewater by Different Algae and Cyanobacteria. Algae and Sustainable Technologies.

[49] Sánchez-Bayo A., Morales V., Rodríguez R., Vicente G., Bautista L.F. (2020). Cultivation of Microalgae and Cyanobacteria: Effect of Operating Conditions on Growth and Biomass Composition. Molecules.

[50] El-Bestawy E. (2008). Treatment of Mixed Domestic–Industrial Wastewater Using Cyanobacteria. J. Ind. Microbiol. Biotechnol..

[51] Eriksen N.T. (2008). Production of Phycocyanin—A Pigment with Applications in Biology, Biotechnology, Foods and Medicine. Appl. Microbiol. Biotechnol..

[52] Winayu B.N.R., Ho J.-Y., Hsueh H.-T., Chu H. (2025). Multifunctional *Thermosynechococcus* sp. CL-1 Cultivation in Swine Wastewater for Nutrients Utilization, CO_2_ Fixation, and C-Phycocyanin Production. J. Taiwan Inst. Chem. Eng..

[53] Erratt K.J., Creed I.F., Trick C.G. (2018). Comparative Effects of Ammonium, Nitrate and Urea on Growth and Photosynthetic Efficiency of Three Bloom-Forming Cyanobacteria. Freshw. Biol..

[54] González A., Bes M.T., Valladares A., Peleato M.L., Fillat M.F. (2012). FurA Is the Master Regulator of Iron Homeostasis and Modulates the Expression of Tetrapyrrole Biosynthesis Genes in *Anabaena* sp. PCC 7120. Environ. Microbiol..

