# Supplementary material for: Bioremediation Potential of a Non-Axenic Cyanobacterium Synechococcus sp. for Municipal Wastewater Treatment in the Peruvian Amazon: Growth Kinetics, Ammonium Removal, and Biochemical Characterization Within a Circular Bioeconomy Framework
Source: BioTech (Basel). 2025 May 13;14(2):36. doi: 10.3390/biotech14020036 (PMC12101411; doi:10.3390/biotech14020036)
Supplement: Supplementary file 1 [file biotech-14-00036-s001.zip › biotech-3495327-supplementary.pdf]

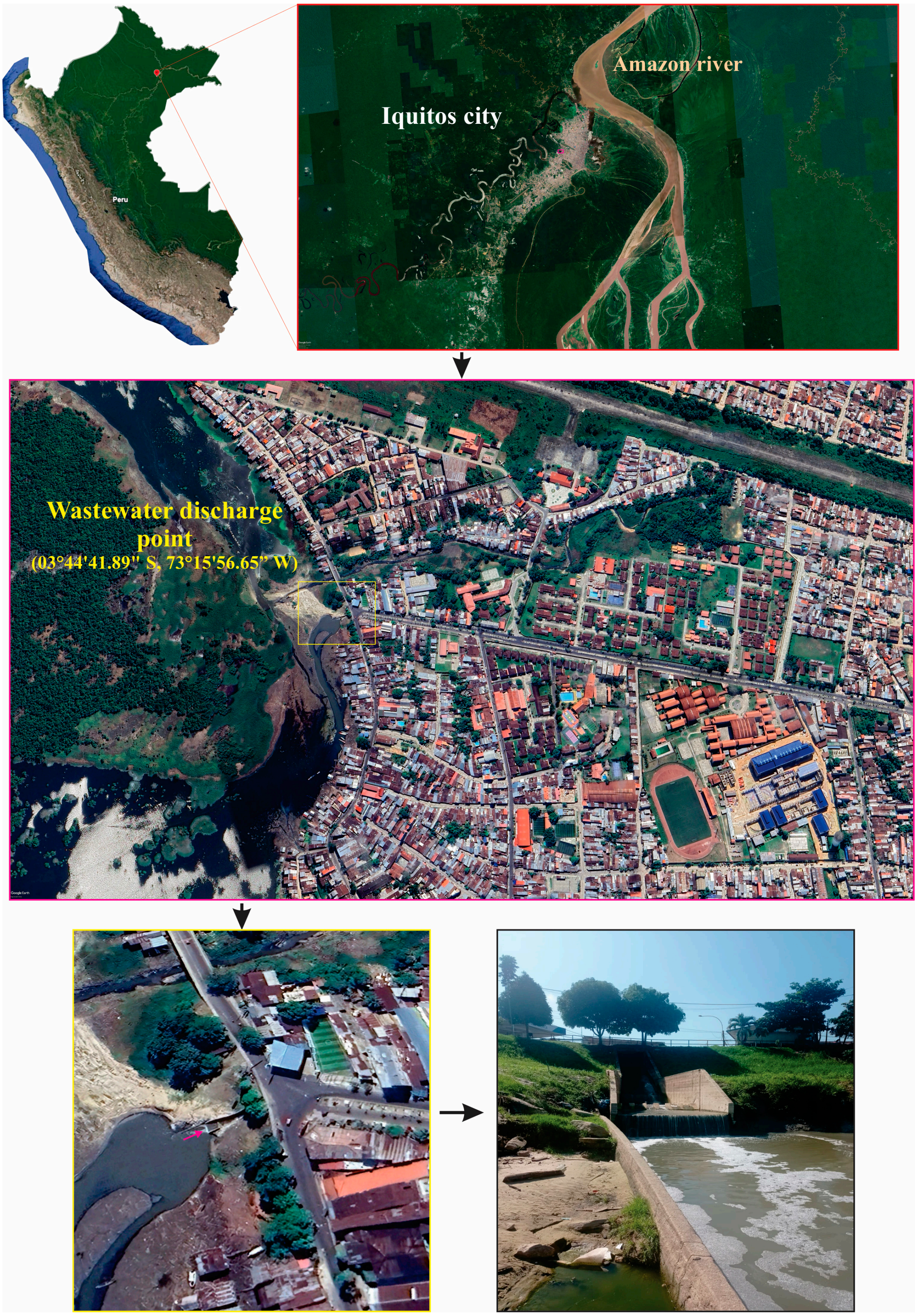

Figure S1. Geographic location and aerial views of the municipal wastewater discharge point in Moronacocha Lake, Iquitos, Peru. Top: Location of Iquitos city in northeastern Peru and its position relative to the Amazon River. Middle: Satellite image of the urban area showing the wastewater discharge point (03°44′41.89″ S, 73°15′56.65″ W). Bottom: Detailed aerial view and ground-level photograph of the discharge infrastructure where wastewater samples were collected for this study.

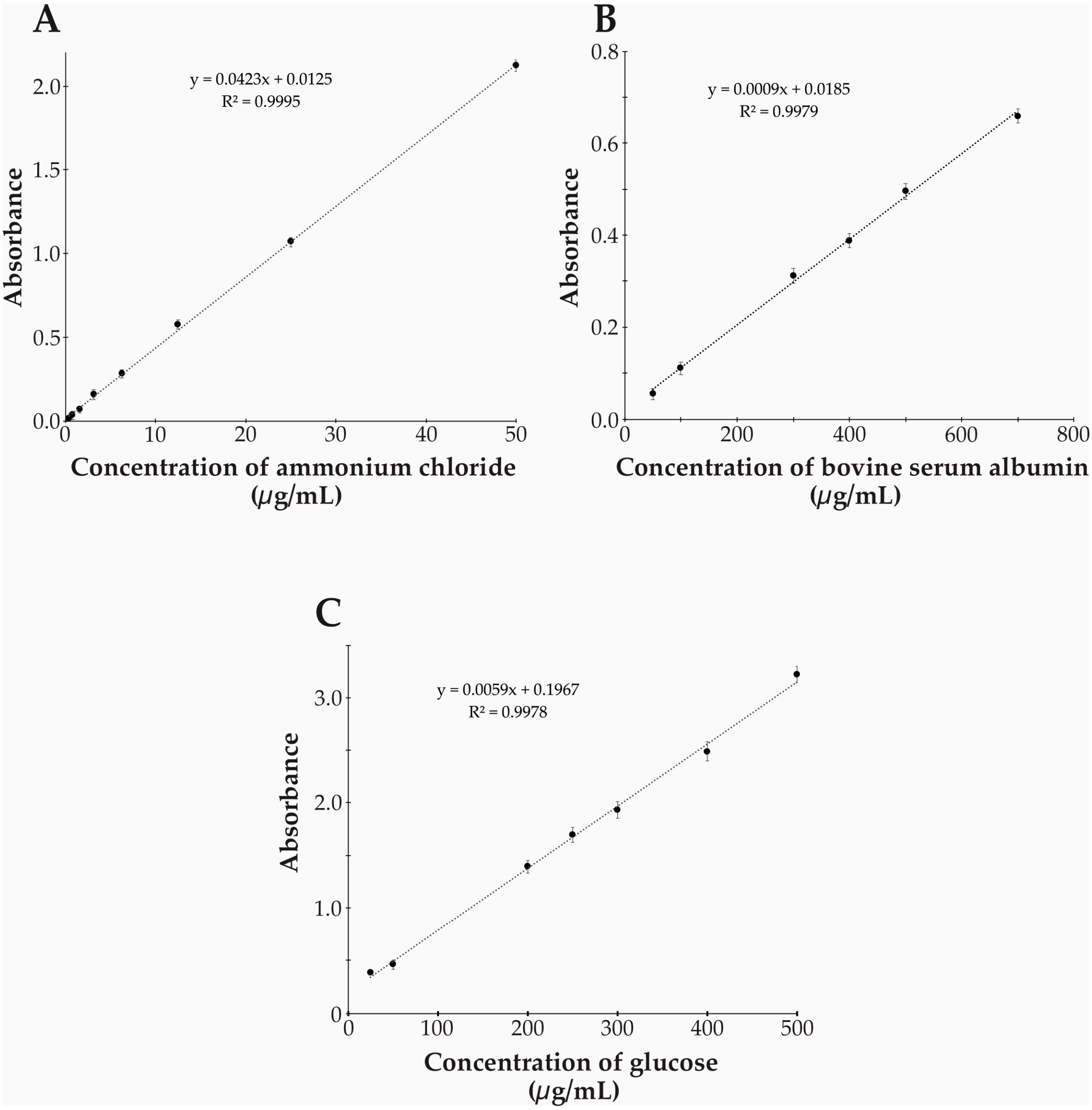

Figure S2. Standard calibration curves for analytical measurements used in this study. (A) Ammonium chloride calibration curve (indophenol blue method at 640 nm) showing linear relationship (R^2 = 0.9995) used for quantifying ammonium in wastewater treatments; (B) Bovine serum albumin calibration curve (R^2 = 0.9979) used for protein content determination in cyanobacterial biomass; (C) Glucose calibration curve (R^2 = 0.9978) used for carbohydrate content determination in cyanobacterial biomass. Each calibration curve demonstrates high linearity with correlation coefficients exceeding 0.995, ensuring reliable quantification of respective compounds. Linear regression equations and correlation coefficients (R^2) are shown for each calibration curve.

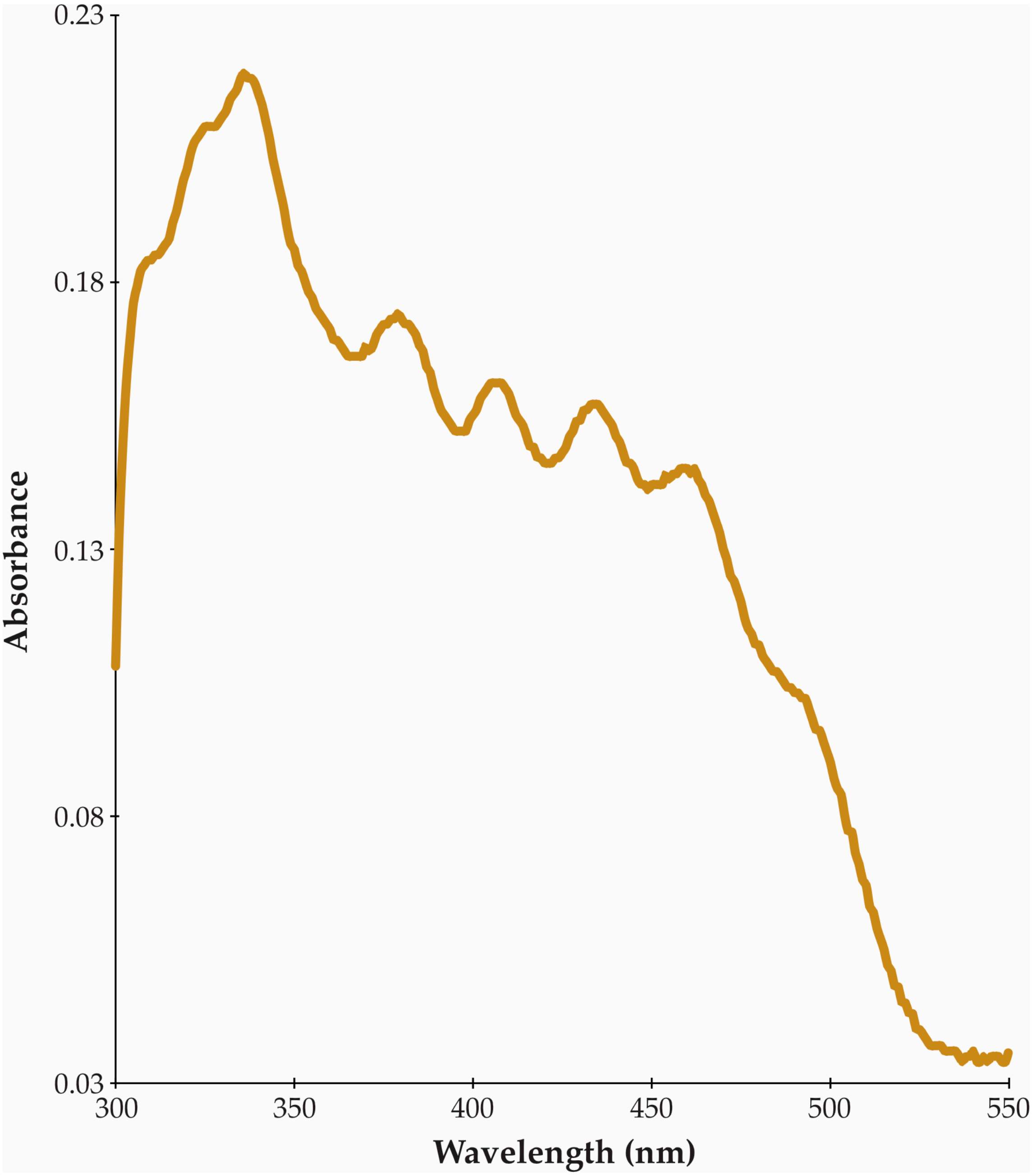

Figure S3. Absorption spectrum of carotenoids extracted from *Synechococcus* sp. biomass. The spectrum shows a characteristic absorbance pattern of carotenoids with an absorption maximum around 330–340 nm and multiple smaller peaks corresponding to vibrational fine structure. This reference spectrum was used for identification and relative quantification of carotenoid content in cyanobacterial biomass grown under different wastewater treatments.

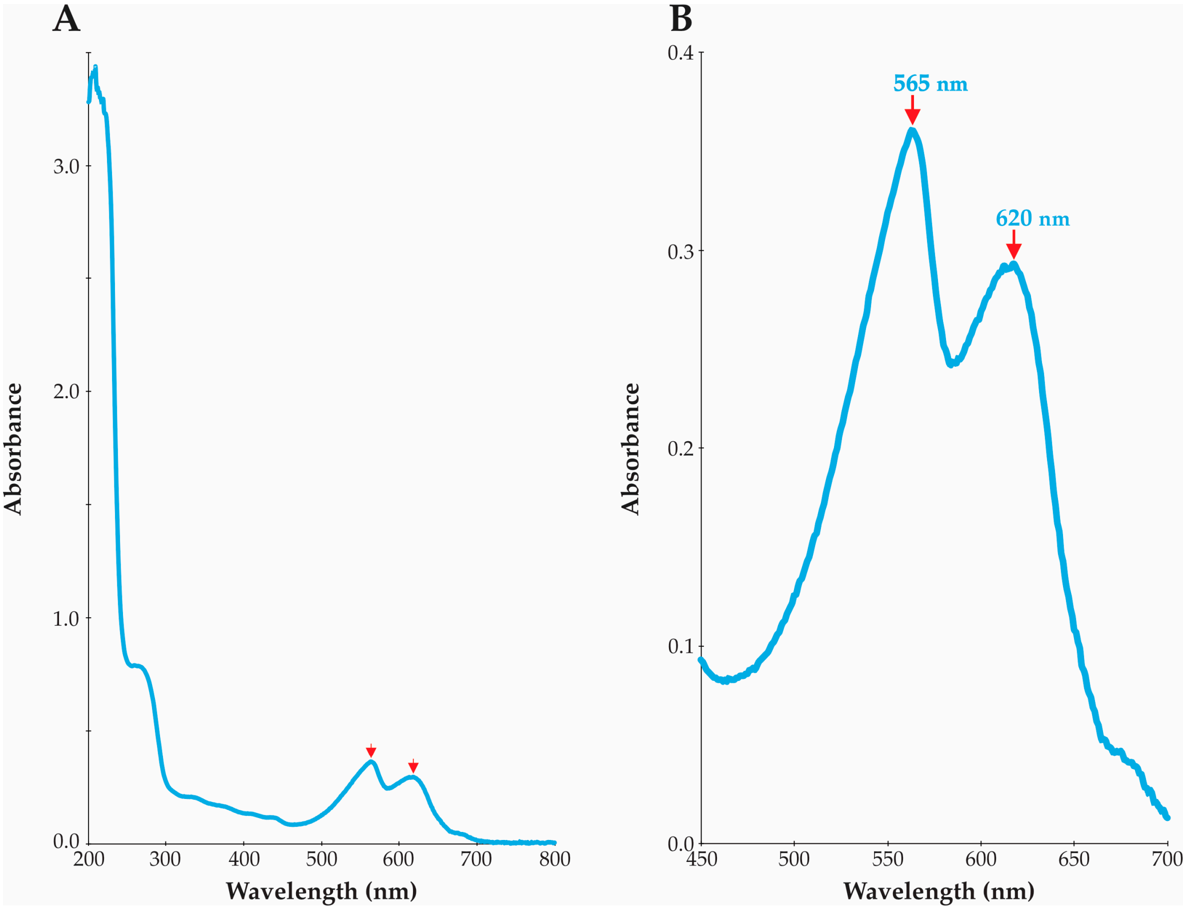

Figure S4. Absorption spectra of phycobiliproteins extracted from *Synechococcus* sp. biomass. (A) Full spectral scan (200–800 nm) of partially purified phycocyanin showing characteristic peaks in the 500–650 nm region; (B) Detailed absorption profile of phycobiliproteins in the visible region (450–700 nm) with characteristic absorption maxima at 565 nm and 620 nm, corresponding to phycoerythrin and phycocyanin, respectively. These spectra were used for pigment identification and quantification in cyanobacterial biomass grown under different wastewater treatments.
